# Forensic Analysis and Identification Processes in Mass Disasters: Explosion of Gun Powder in the Fireworks Factory

**DOI:** 10.3390/molecules27010244

**Published:** 2021-12-31

**Authors:** Maricla Marrone, Francesca Tarantino, Alessandra Stellacci, Stefania Lonero Baldassarra, Gerardo Cazzato, Francesco Vinci, Alessandro Dell’Erba

**Affiliations:** 1 Section of Legal Medicine, Department of Interdisciplinary Medicine, University of Bari “Aldo Moro”, 70124 Bari, Italy; mariclamarrone@hotmail.it (M.M.); tarantinofrancesca.ft@gmail.com (F.T.); alestellacci@gmail.com (A.S.); stefania.lonerobaldassarra@uniba.it (S.L.B.); francesco.vinci@uniba.it (F.V.); alessandro.dellerba@uniba.it (A.D.); 2 Section of Molecular Pathology, Department of Emergency and Organ Transplantation (DETO), University of Bari “Aldo Moro”, 70124 Bari, Italy

**Keywords:** forensic analyzes, DNA identification, mass disasters, reconstruction

## Abstract

A mass disaster is a situation that involves criticality between the number of victims and resources, in terms of both men and means, present on the site of an event that is mostly unexpected and sudden. In the multidisciplinary teams that intervene, the role of forensic pathologists, who are responsible for the direction and coordination of post-mortem operations, is central, and must remain so. The authors report the case of an explosion of a pyrotechnic artifice factory, as a result of which numerous victims and injuries are recorded. So, the team completed the autopsies and created a protocol to obtain biological samples (bones, blood, teeth, muscles), while the forensic pathologists contacted the families of the alleged victims and each provided a blood sample that was collected for the DNA. The geneticist, using the method of gene extraction and amplification, obtained the DNA from each bone, tooth, and muscle of blood taken from the victims and then compared it with that extracted from the blood samples of the relatives; the electropherograms showed at least one allele for each genetic marker of the “Combined DNA Index System” in common between the victims and the families, thus allowing to establish the identity of all the subjects involved in the event. Having established the identity of all workers, it was possible to determine their whereabouts in the environment at the time of the location of fires and explosions. The results of the various forensic analyzes (autopsies, genetic investigations and even traumatological investigations) have allowed us to validate a scientific method useful in all mass disasters even when any type of anthropological or forensic dental research is difficult.

## 1. Introduction

The succession of natural catastrophic events (tsunamis and hurricanes), of terrorist attacks (11 September 2001 in New York, 11 March 2003 in Madrid and London, 13 November 2015 in Paris), air accidents and events related to the increasing frequency of use of industrial and military artifices, have determined the most urgent need to activate particular procedures aimed at solving problems of a medico-legal and pathological forensic nature [1,2,3,4,5,6,7,8,9,10,11,12]. One of the most commonly accepted definitions is that a mass disaster is a situation that involves criticality between the number of victims and resources, in terms of both men and means, present on the site of the event which is mostly unexpected and sudden [13].

**Figure 1:**
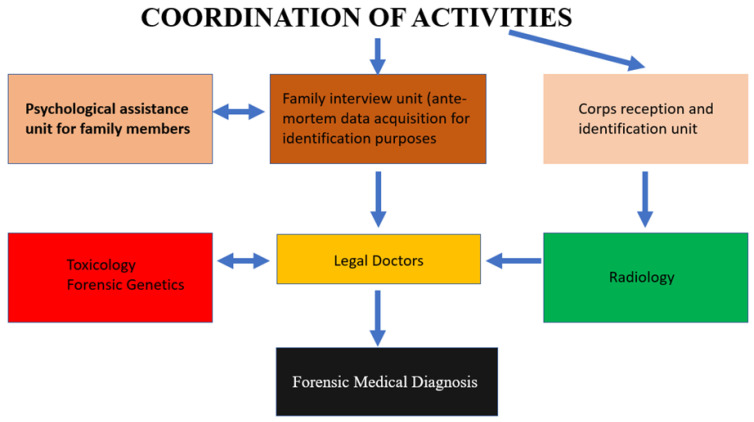
Flow-chart of actions to be taken in the course of a mass disaster.

On this basis, it appears necessary that even forensic medicine should provide for an organization to deal with the aforementioned situations [14]. In the multidisciplinary teams that intervene, the role of forensic pathologists, who are responsible for the direction and coordination of post-mortem operations, is central, and must remain so [15]. In fact, when considering the concrete problems that arise in mass disasters—first of all—the identifying one, that of the study of harm and the reconstruction of the dynamics of the fact for judicial purposes, cannot fail to highlight the centrality of the figure of the coroner [6]. They must in fact be able to deal with different situations from the point of view of cases with the presence of multiform and multicentric situations that are harmful, to each of which must be attributed the exact diagnostic-nosographic position from which it will not be possible, regardless, for a correct and exact overall framework of the case [16,17,18]. To this end, the uniformity of the procedures adopted with homogeneity in the acquisition of data and biological samples is fundamental, guaranteeing a minimum standard and quality verification of the performance [19] (Figure 1).

## 2. Case Report

On 24 July 2015, at around 12.30, a pyrotechnic artifice factory located in Bari exploded, injuring and partially burying the people employed there under the rubble. Rescue operations began shortly thereafter, which proceeded, in the following hours, to become the recovery of the wounded who were promptly transported to the health facilities of Bari, Brindisi and Naples, as well as numerous bodies that were gradually brought to the morgue and the Forensic Medicine Section of the Bari Polyclinic. The fire went on for a long time, hindering rescue operations. Overall, there were 10 victims of the explosion. The last victim died five days after the explosion, at an off-site hospital (which will not be described in the following discussion). The suspected causes, initially, were the most varied, with possibilities ranging from random accidents, to human errors up to possible terrorist attacks, especially given the social context present at that time.

## 3. Materials and Methods

On 24 July 2015, around 7.00 pm, forensic pathologists arrived at the Mortuary of the Policlinico di Bari n. 5 blue canvas bags and n. 10 light blue plastic bags marked with the numbers 1, 2, 3, 4, 5, 6, 7, 8, 9, 10, 12, 13, 15, 16 and 17. The numbering of the bags was given by the rescuers, the missing numbers (not reached in the morgue) probably contained non-biological material. The necropsy operations made it possible to detect the following:

- Sack n. 1 containing: a decayed corpse cataloged as CORPSE SACK n. 1; a head cataloged as SACK HEAD n. 1 bis;
- Bag n. 2 containing: an extensively broken corpse, consisting in principle of the left hemisome (part of the half face, neck, upper limb, part of the trunk, lower limb up to the distal portion of the femur), cataloged as CORPSE BAG n. 2;
- Bag n. 3 containing: soft tissue, referable in the first instance to a portion of the gastro-enteric system, cataloged as BAG n. 3;
- Bag n. 4 containing: soft tissue with a parenchymatous consistency, 20 × 12 cm in size, referable in the first instance to the spleen, cataloged as BAG n. 4;
- Bag n. 5 containing: soft tissue with dimensions of 17 × 8.5 cm cataloged as BAG n. 5;
- Bag n. 6 containing: a corpse cataloged as CORPSE SACK n. 6;
- Bag n. 7 containing: a charred corpse cataloged as CORPSE SACK n. 7;
- Bag n. 8 containing: part of the right lower limb (proximal third of leg and foot) cataloged as FOOT BAG n. 8;
- Bag n. 9 containing: soft tissue and bone tissue, referable in the first instance to a hemibacin, cataloged as BAG n. 9;
- Bag n. 10 containing: some carbonized vertebral elements, referable to as thoracic vertebrae; a left foot 23.5 cm long cataloged as FOOT BAG n.10;
- Bag n. 12 containing: a right foot 31 cm long cataloged as FOOT BAG n. 12;
- Bag n. 13 containing: soft tissue and bone, referable in the first instance to the left hemibacin, with extensively charred stump of the femur, cataloged as BAG n. 13;
- Bag n. 15 containing: soft tissue and bone tissue, referable in the first instance to a hemibacin, cataloged as BAG n. 15;
- Bag n. 16 containing: a charred corpse cataloged as CORPSE SACK n. 16;
- Bag n. 17 containing: soft tissue and bone tissue, referable in the first instance to the scapulohumeral joint, cataloged as BAG n. 17.

In the following three days, three bodies of severely burned subjects arrived at the Forensic Medicine Section of Bari, previously hospitalized in the Intensive Care Department (two of the Bari Polyclinic and one of Brindisi), were cataloged by us as BURNED n. 2, BURNED n. 3 and BURNED TOAST. Finally, on 29 July 2015, bag n. 14, containing a broken corpse and in an advanced state of putrefaction, was cataloged as CORPSE SACCA n. 14. At the end of the preliminary medical-legal operations, the total number of victims was nine, to be identified with certainty from a list of “missing” people, relating to the place of the event. There were, therefore, nine male subjects, aged between 20 and 69 years; in all cases an autopsy was performed. Histological samples were taken and subsequently examined under an optical microscope. During the autopsy examination, during the examination of the airways, traces of soot were found in the bodies that initially survived the explosion, and this provided details to estimate with a better degree of certainty the time of death. During the judicial inspection, carried out on the following 6 August at the pyrotechnic artifice factory, further biological samples were found, indicated as follows:

- charred vertebral elements, referable to as thoraco-lumbar vertebrae and a complex of charred long bones surrounded by soft tissue, referable to as fragment of the thoracic cage (ribs), cataloged as SACK n. 21;
- right upper limb, consisting of humerus (distal third), elbow joint, soft and bone tissues of the forearm and ipsilateral hand, cataloged as BAG n. 22;
- a fragment of charred soft tissue, apparently referable to as muscle tissue and fragments of long bones, cataloged as SACK n. 23;
- fragment of the right hemimandible (still including some dental elements), with carbonization of the soft tissues, cataloged as SACK n. 24;
- soft tissue, referable to in the first instance as muscle tissue, extensively charred, and a fragment of long bone, referable to in the first instance as a section of the femur, cataloged as BAG n. 26;
- a fragment of long bone, referable to in the first instance as a section of the femur, cataloged as SACK n. 27.

At the end of the collection, the aforementioned bags were stored in a suitable environment at the Forensic Medicine Section of the Bari Polyclinic (cold room) and then their contents were subsequently examined; biological samples necessary for genetic investigations were taken from each bag. During the examinations of the corpses and corpse remains, suitable samples were taken for genetic typing aimed at identification (blood, muscle, teeth and bones). These samples were used comparatively with other samples taken and collected from the families of the victims, as well as, in the absence of these, from material obtained from personal objects of common use certainly belonging to some of the subjects involved in the explosion and provided by trusted defenders of these. In particular, the investigations carried out on the aforementioned samples were conducted with the aim of obtaining the genetic profiles useful for comparison with those of the presumed family members and personal objects belonging to the victims. The reference samples consisted of blood taken from first degree relatives (mother, father and brothers) for the victims of Italian nationality, as well as razors and toothbrushes, provided by the lawyers of the alleged non-EU victims.

On the findings listed below, DNA typing was performed for the genetic polymorphisms present on the autosomal chromosomes D8S1179—D21S11—D7S820—CSF1P0—D3S1358—TH01—D13S317—D16S539 D2S1338—D19S433—VWA—TPOX—D18S51—D5S818—FGA and AMELOGENINE SYSTEMS.

The typing of genetic polymorphisms present on chromosome Y was performed exclusively on the specimens classified as F1 and F2 specimens: DYS391—DYS389 I—DYS439—DYS389 II—DYS393—DYS390—DYS385—DYS438—DYS437—DYS19—DYS392—DYS456—DYS456—DYS635—Y-GATA -DYS448.

### 3.1. DNA Extraction from Blood Samples

The blood samples were fixed on different Indicating FTA^®^ Mini Cards (Whatman^®^) and, subsequently, DNA extraction was performed using an aliquot of each sample and the FTA Purification Reagent (Whatman^®^) following the specific protocol for the extraction of DNA from blood samples indicated by the manufacturer.

### 3.2. Dna Extraction from Bone Fragments and Teeth

500 milligrams of powder of each bone and the individual teeth were subjected to DNA extraction performed using both the commercial NucleoSpin^®^ DNA Trace kit and NucleoSpin^®^ DNA Tissue both from the company Macherey-Nagel, following a modified protocol for the bones (Piglionica et al., 2012), and for the teeth the protocol indicated by the manufacturer for DNA extraction from tissues.

### 3.3. Dna Extraction from Carbonized Tissues

An aliquot of each carbonized tissue was subjected to DNA extraction performed using the commercial kit NucleoSpin^®^ DNA Tissue from the company Macherey-Nagel, following the specific protocol for the isolation of genomic DNA from tissues indicated by the manufacturer.

### 3.4. Dna Extraction from the Toothbrush and Razor

The toothbrush and the razor were subjected to DNA extraction using the QIAamp^®^ Investigator kit from Qiagen following the specific protocol for small traces of saliva.

The DNA extracted from Samples D4, D5, D6, D6B, D7, G5, H3, I1, I2 and L was concentrated and purified using Amicon “Microcon 100” micro concentrators and quantized using an Eppendorf BioPhotometer Plus spectrophotometer; the results were as follows:

1. DNA Sample D4 = 7 ng/µL
2. DNA Sample D5 = 4.2 ng/µL
3. DNA Sample D6 = 3 ng/µL
4. DNA Sample D6B = 5.3 ng/µL
5. DNA Sample D7 = 2.1 ng/µL
6. DNA Sample G5 = 5.5 ng/µL
7. DNA Sample H3 = 2.1 ng/µL
8. DNA Sample I1 = 3.5 ng/µL
9. DNA Sample I2 = 3.8 ng/µL
10. DNA Sample L = 2.3 ng/µL

DNA TYPING: D8S1179, D21S11, D7S820, CSF1PO, D3S1358, TH01, D13S317, D16S539, D2S1338, D19S433, VWA, TPOX, D18S51, D5S818, FGA AND AMELOGENINE SYSTEMS.

The amplification of loci D8S1179, D21S11, D7S820, CSF1PO, D3S1358, TH01, D13S317, D16S539, D2S1338, D19S433, vWa, TPOX, D18S51, D5S818, FGA and Amelogenin was performed in a multiplex reaction using the multiplex reagents AmpFlSTR^®^ Identifiler^®^ Plus PCR Amplification Kit” by Applied Biosystems, in accordance with the instructions of the manufacturer, in a thermal cycler model GeneAmp^®^ PCR System 9700 Applied Biosystem. For each PCR, 3 ng of DNA were used in a reaction volume of 25 µL. After amplification, an aliquot of each sample was subjected to capillary electrophoresis in an ABI PRISM^®^ 310 Genetic Analyzer, an Applied Biosystem automatic sequencer. The results of the typing of the genetic characteristics are reported in the following tables.

DNA TYPING: DYS456, DYS389I, DYS390, DYS389II, DYS458, DYS19, DYS385, DYS393, DYS391, DYS439, DYS635, DYS392, Y-GATA, DYS437, DYS438, DYS448 SYSTEMS. The amplification of the loci DYS456, DYS389I, DYS390, DYS389II, DYS458, DYS19, DYS385, DYS393, DYS391, DYS439, DYS635, DYS392, Y-GATA, DYS437, DYS438, DYS448 was performed in a multiplex reagent amplification reaction of the “AmpFlSTR^®^Yfiler™” kit by Applied Biosystem, in accordance with the instructions of the manufacturer, in a thermal cycler model GeneAmp^®^ PCR System 9700, Applied Biosystem. For each PCR, 3 ng of DNA were used in a reaction volume of 25 µL. After amplification, an aliquot of each sample was subjected to capillary electrophoresis in an ABI PRISM^®^310 Genetic Analyzer, an Applied Biosystem automatic sequencer. The results of the typing of genetic characteristics are shown in the following table.

The typing of the genetic markers present on the autosomal chromosomes performed on the samples C1, C2, D2, D4, D5, D6, D6B, D7, E1, E2, F1, F2, G1, G2, G3, G5, H3, H1 and I2 gave a positive result for all sixteen loci analyzed (Table 1, Table 2 and Table 3). For the same markers, samples I1 (humerus fragment) and L (tooth) gave positive results, respectively, for 12 out of 16 and 13 out of 16 analyzed loci (Table 4). The typing of the genetic markers present on the Y chromosome of the F1 and F2 samples gave a positive response respectively for 8 out of 16 and 15 out of 16 analyzed loci (Table 5).

The cause of the lack of amplification of some genetic loci was to be found in the insufficient and/or probably degraded quantity of DNA or in the probable presence of inhibitors of the amplification reaction. The biological samples of the corpses and human remains found during the inspections were compared with the blood samples of the presumed relatives or with biological material present on objects that belonged in life to the bodies to be identified. The results showed compatibility in terms of familiarity with the comparison materials; so much of which has allowed the identification of the victims. Based on the analyses carried out, it was also possible to go back to the time of death. In the case in question, the evaluation of the classic thanato-chronological parameters had to take into account historical and circumstantial data.

In fact, the effects of greater intensity related to the proximity to the epicenter of the explosion amply justified the immediate death, which, therefore, coincided with the time established in the acts in which the explosion occurred. In any case, it is emphasized that the chronological data observed for each individual body did not contrast with this hypothesis. This concerns the corpses of five victims. As for the three burned subjects, the death of these occurred in a hospital environment; and, therefore, the moment of death was thus documented in the medical record. With regard to the last body, however, since it was a charred but not decayed corpse, there were no elements to establish whether the death occurred at the same time as the explosion or occurred later due to the development of the fire. In other words, it was possible to hypothesize, even though there could be no criterion of certainty, a survival, in any case in our opinion, limited. We recall that only 1 of the 10 victims was not analyzed by the authors, as it was transported to a hospital outside the region.

Based on the characteristics of the injuries found on the corpses, it was finally possible to hypothesize the location of each victim at the time of the explosion, relating it to the site where the remains were found:

- four bodies showed injuries directly related to the explosive effect of the explosion, with death coming instantly;
- one corpse was instead characterized by an injury more related to blunt and compressive effects related to the collapse of one of the building structures present in the place of the accident and in which the victim was at the time of the explosion;
- the other victims, on the other hand, died as a result of burns reported and involving 100% of the body surface; this is also assuming, for one of these, the recurrence of the action of minor toxic and/or contusive phenomena. We carried out histopathological examinations that showed dermo-epidermal damage extended to the skin surface, with deposition of exogenous blackish pigment. Furthermore, we highlighted vacuolation of basal keratinocytes, epidermal damage with exulceration, progressive hyalinization of the dermal collagen fibers and coarctation of the samples [20,21]. With the support of histology and after excluding other causes, we had confirmation that it was already visible at macroscopic external inspection.

## 4. Discussion and Conclusions

In forensic medicine, one of the most difficult problems is, relating to the identification of a large number of subjects involved in a mass disaster, closely connected with their position in the environment at the time the event occurs and with the need to make hypotheses reconstructive [22,23,24].

In this regard, the lack of ante-mortem data, especially of the non-EU individuals (as in the case under study), and the scarcity of post-mortem data (detectable on cadavers), often lead to not allowing identification of the subjects with the techniques alone. forensic anthropological and odontological [25,26,27,28]. The type of mass disaster, in particular, constitutes an element capable of varying to a more or less marked extent the characteristics and any marks that characterize an individual compared to another, making the recognition, albeit “indirect”, of the victims even less easy [29].

Therefore, upon the occurrence of such a phenomenon, the application of forensic genetics techniques are of “mandatory” use in order to be able to give an identity to the victims involved, to be able to recompose the deceased corpses and, therefore, to be able to reconstruct the events more corresponding to reality; this is based on the location of the corpses and their fragments in the environment, identifying the time of death, and also in light of the thanato-chronological parameters [11,30,31,32]. The limitations of genetics, relating to the possibility of using degraded material or small quantities of samples on which to perform DNA extraction and typing, now appear to be outdated in most of the samples analyzed [33].

It is in fact always possible to type an individual from blood, or from objects that belonged to them in life, yet also, in most cases, from charred tissues and from bone and dental matrices, which are the best preserved in all types of mass disasters.

Ultimately, if we want to limit the scope of the assessment here to charred and broken corpses, individual identification must, therefore, take into account the different substrates used, the main ones being charred tissue and “cooked” blood.

The method applied by the authors is an attempt to standardize an undoubtedly difficult evaluation that can often suffer, as already mentioned, from the absence of factors that are often not easy to find and not always assessable (acquisition of ante-mortem data and comparison with post-mortem data). However, the results obtained, although relating to only seven cases (the only ones to be identified), seem to confirm the findings made on the basis of the other elements currently used in the medical-legal field for the reconstruction of events [34].

Finally, the medico-legal reconstruction and the investigators made it possible to trace the true causes of the mass disaster, which corresponded to an excessive amount of gunpowder, from the use of tools not suitable for cutting explosive substances, as well as the lack of some of the most important safety points such as the presence of fireproof suits. All this made it clear that it was a disaster of incompetence rather than a terrorist attack.

## Figures and Tables

**Table 1:** Summary of the samples taken and used in the work.

Samples Taken from Corpses or from Bags Containing Biological Remains	Samples Taken from Relatives of the Degree or from Objects Belonging to the Victims
Sample C1 Blood corpse Burned n. 3 (P.R.)	Sample C2 Blood P. A. presumed corpse mother Burned n. 3
Sample D4 Tooth 41 Cadaver Bag 2 (P.M.)	Sample D2 Blood B. R. presumed mother corpse Bag 2
Sample D5 Tooth 13 cadaver Sacca n. 1 bis (P.M.)	
Sample D6 Corpse foot fragment Bag 8 (P.M.)	
Sample D6B Corpse foot fragment Bag n. 10 (P.M.)	
Sample D7 Fragment of humerus corpse Sacca n. 22 (P.M.)	
Sample E1 Corpse blood Bag 6 (P.G.)	Sample E2 Blood F.E. presumed corpse mother Bag 6
Sample F1 Blood corpse Bag n.16 (D.C.V.)	Sample F2 Blood D.C.E. alleged corpse brother Bag 16
Sample G1 Corpse blood Bag 1 (N.K.P.)	Sample G2 Blood presumed corpse mother Bag 1 (K.D.)
	Sample G3 Blood presumed corpse father Bag n. 1 (R.)
Sample G5 Foot fragment Bag 12 (B. H.)	Sample H3 Toothbrush B.H.
Sample I1 Fragment of humerus corpse Bag 14 (B.H.)	
Sample L Tooth 48 Bag 24 (B. H.)	
Sample H1 Corpse blood Bag n. 7 (M. S.)	Sample I2 razor blade M.S.

**Table 2:** Autosomal DNA chromosome polymorphisms results.

Genetic Markers	Sample Genotype C1	Sample Genotype C2	Sample Genotype D2	Sample Genotype D4	Sample Genotype D5	Sample Genotype D6	Sample Genotype D6B	Sample Genotype D7
D8S1179	13/14	13/14	8/16	8/13	8/13	8/13	8/13	8/13
D21S11	29/30	29/29	31.2/31.2	31.2/32.2	31.2/32.2	31.2/32.2	31.2/32.2	31.2/32.2
D7S820	9/12	9/11	10/10	10/10	10/10	10/10	10/10	10/10
CSF1P0	10/12	10/10	10/13	10/10	10/10	10/10	10/10	10/10
D3S1358	15/16	14/15	18/18	17/18	17/18	17/18	17/18	17/18
TH01	6/10	9/10	8/9	8/9	8/9	8/9	8/9	8/9
D13S317	12/12	11/12	9/10	10/12	10/12	10/12	10/12	10/12
D16S539	12/13	11/13	11/13	9/11	9/11	9/11	9/11	9/11
D2S1338	17/23	17/25	17/25	24/25	24/25	24/25	24/25	24/25
D19S433	13/14	13/14	14/15	15/15	15/15	15/15	15/15	15/15
VWA	15/17	17/18	17/17	16/17	16/17	16/17	16/17	16/17
TPOX	9/11	8/11	8/11	8/11	8/11	8/11	8/11	8/11
D18S51	13/14	14/16	13/16	16/16	16/16	16/16	16/16	16/16
D5S818	9/11	11/13	10/12	12/13	12/13	12/13	12/13	12/13
FGA	21/22	22/23	23/24	23/23	23/23	23/23	23/23	23/23
Amelogenina	X/Y	X/X	X/X	X/Y	X/Y	X/Y	X/Y	X/Y

**Table 3:** Autosomal DNA chromosome polymorphisms results.

Genetic Markers	Sample Genotype E1	Sample Genotype E2	Sample Genotype F1	Sample Genotype F2	Sample Genotype G1	Sample Genotype G2	Sample Genotype G3
D8S1179	13/15	13/13	11/14	14/14	12/14	12/14	12/14
D21S11	28/30.2	28/30.2	27/29	27/32	29/31.2	28/29	31.2/32.2
D7S820	10/11	8/10	10/10	10/11	8/11	8/11	8/10
CSF1P0	10/12	11/12	10/12	10/12	7/11	7/12	10/11
D3S1358	17/18	16/18	16/18	17/18	15/17	15/18	15/17
TH01	6/9	6/9	6/6	6/7	6/7	6/6	7/8
D13S317	10/10	9/10	8/12	11/12	11/12	12/12	11/12
D16S539	11/12	11/12	12/12	12/12	11/12	11/12	11/13
D2S1338	16/18	18/19	17/22	17/22	18/20	18/18	19/20
D19S433	13/16	14.2/16	12/14	12/14	13/14	13/14	14/14
VWA	16/16	16/17	16/19	14/16	17/19	16/19	16/17
TPOX	8/9	9/10	8/9	8/9	10/11	11/12	10/12
D18S51	14/19	12/14	15/16	15/16	15/16	16/16	15/19
D5S818	10/11	10/11	12/13	12/12	10/12	12/12	10/11
FGA	19/25	19/21	22/24	20/20	20/23	23/26	20/25
Amelogenina	X/Y	X/Y	X/Y	X/Y	X/Y	X/X	X/Y

**Table 4:** Autosomal DNA chromosome polymorphisms results.

Genetic Markers	Sample Genotype G5	Sample Genotype H3	Sample Genotype I1	Sample Genotype L	Sample Genotype H1	Sample Genotype I2
D8S1179	10/15	10/15	10/15	10/15	11/14	11/14
D21S11	28/31.2	28/31.2	28/31.2	28/31.2	28/29	28/29
D7S820	12/13	12/13	//	//	9/9	9/9
CSF1P0	11/11	11/11	//	//	11/12	11/12
D3S1358	15/19	15/19	15/19	15/19	16/18	16/1
TH01	6/9	6/9	6/9	6/9	9.3/9.3	9.3/9.3
D13S317	12/12	12/12	12/12	12/12	12/12	12/12
D16S539	9/12	9/12	9/12	9/12	11/11	11/11
D2S1338	19/19	19/19	19/19	19/19	17/17	17/17
D19S433	12.2/14	12.2/14	12.2/14	12.2/14	15.2/16.2	15.2/16.2
VWA	15/17	15/17	15/17	15/17	17/17	17/17
TPOX	8/9	8/9	8/9	8/9	11/11	11/11
D18S51	16/18	16/18	//	//	16/16	16/16
D5S818	11/11	11/11	11/11	11/11	11/13	11/13
FGA	20/22	20/22	//	20/22	23/23	23/23
Amelogenina	X/Y	X/Y	X/Y	X/Y	X/Y	X/Y

**Table 5:** Y chromosome genetic marker results.

Genetic Markers	Sample F1	Sample F2
DYS456	15	15
DYS389I	12	12
DYS390	22	22
DYS389II	//	29
DYS458	//	//
DYS19	//	13
DYS385	//	12.15
DYS393	11	11
DYS391	11	11
DYS439	//	14
DYS635	21	21
DYS392	//	16
Y-GATA	11	11
DYS437	15	15
DYS438	//	10
DYS448	//	19

## Data Availability

Not applicable.

## Sample Availability

Samples of the compounds are not available from the authors.
